# Combined Nimotuzumab with Chemoradiotherapy for Locally Advanced Head and Neck Squamous Cell Carcinoma

**DOI:** 10.7759/cureus.8105

**Published:** 2020-05-13

**Authors:** Nguyen Thi Thai Hoa, Huynh Quang Huy

**Affiliations:** 1 Department of Internal Medicine, Vietnam National Cancer Hospital, Hanoi, VNM; 2 Radiology, Pham Ngoc Thach University of Medicine, Ho Chi Minh City, VNM

**Keywords:** monoclonnal antibody, chemoradiotherapy, response rate, overall survival, progression - free survival

## Abstract

Background

Most head and neck cancers (HNCs), specifically squamous cell carcinoma, express epidermal growth factor and are associated with an inadequate response to radiotherapy and chemotherapy. Anti-epidermal growth factor receptor (EGFR) monoclonal antibodies (mAb) increase response rates and survival when combined with radiotherapy or chemoradiotherapy (CRT). This study evaluates the outcome and toxicity of the nimotuzumab-CRT combination for stage III, IVa, and IVb squamous cell carcinoma of the head and neck.

Methods

Eighty-seven patients with squamous cell carcinoma of the head and neck, stage III, IVa, or IVb were enrolled in a prospective comparative study. The nimotuzumab plus CRT group consisted of patients who received nimotuzumab 200 mg every week for six consecutive weeks chemoradiation therapy; cisplatin 30 mg/m^2^ every week for six weeks; radiotherapy of 2-Gy/fraction, five fractions/week for a total dose of 70 Gy; and neck lymph node invasion prophylaxis at 50 Gy. The CRT alone arm was treated with CRT (without nimotuzumab).

Results

Tumor response rate of 90.6% was achieved in nimotuzumab plus CRT group (complete response: 58.1%), and 70.4% in CRT alone arm (complete response: 38.6%; p=0.029). The lymph node response rate was 83.4% in nimotuzumab plus CRT group (complete response: 46.7%), and 73.0% in CRT group (complete response: 23.0%). The general response rate in nimotuzumab plus CRT group was 86.0% (complete response: 48.8%), and 68.0% in CRT alone arm (complete response: 36.0%). Twelve-month overall survival (OS) was 75.1% for the nimotuzumab plus CRT group and 54.4% for the CRT group. The 24-month survival was 48.0% (nimotuzumab plus CRT group) and 29.0% (CRT alone arm). The median OS was 20 months and 13 months for nimotuzumab plus CRT group and CRT alone arm, respectively. Progression-free survival (PFS) in the nimotuzumab plus CRT group at 12 months and 24 months was 64.2% and 37.4%, respectively. PFS in the CRT group at 12 months and 24 months was 39.5% and 21.3%, respectively. Infusion reaction presented mildly in two of 43 patients in the nimotuzumab plus CRT group, and no shock occurred. Other toxicity occurrences were similar between the two groups, mainly in grade I, II. Skin rash (grade I only) occurred at a rate of 4.7% in the nimotuzumab plus CRT group.

Conclusion

Nimotuzumab in combination with CRT was well tolerated as a treatment program for locally advanced head and neck squamous cell carcinoma.

## Introduction

Head and neck cancer (HNC) is a group of cancers derived from different locations in the upper respiratory and digestive tracts. HNCs account for 10% of all types of cancer. Most malignant tumors of the head and neck are derived from epithelial surfaces, so more than 90% of cases are usually accounted for by squamous cell carcinoma or its variants. Management of HNC depends on the anatomical location and stage of the disease. In the case of locally advanced disease, chemoradiation therapy with cisplatin gives good results under appropriate indications [1].

More than 95% of HNCs, especially squamous cell carcinoma, express epidermal growth factor, and are associated with a poor response to radiotherapy and chemotherapy [2]. Anti-epidermal growth factor receptor (EGFR) monoclonal antibodies (mAb) increase response rates and survival when combined with radiotherapy or chemoradiotherapy(CRT) [3-6].

In Vietnam, the anti-EGFR mAb nimotuzumab has been used in combination with CRT or radiotherapy for the treatment of HNC since 2009; so far, no studies have addressed the effectiveness and safety of this regimen. This study evaluates the outcome and toxicity of the nimotuzumab-CRT combination for stage III, IVa, and IVb squamous cell carcinoma of the head and neck.

## Materials and methods

Patients

Patients diagnosed with squamous cell carcinoma of the head and neck with the American Joint Committee on Cancer (AJCC) stage III, IVa, or IVb were enrolled in a prospective comparative study [7]. This study was conducted at Vietnam National Cancer Hospital from June 2010 to June 2013 under the approval of the Hospital Ethics Committee (reference number: 01062010/VNCH). Written informed consent was obtained from all patients before inclusion in the study.

Inclusion criteria were as follows: squamous cell carcinoma of the oral cavity, oral pharynx, hypopharynx and larynx (stage III, IVA, or IVB) according to the AJCC; age older than 18 years; first-time treatment; performance status (PS) of zero to two; provided informed consent to participate in the study [7].

Exclusion criteria were as follows: cancer of the salivary glands, sinuses, nasopharynx, skin, or lips; indication for radical surgery; a history of other cancers; a history of chemotherapy, radiotherapy or anti-EGFR treatment; a history of severe chronic diseases (e.g., diabetes, hypertension, HIV, viral hepatitis) resulting in contraindication for chemotherapy or anti-EGFR mAb; and pregnant or breastfeeding women.

Variables of patients were collected, including diagnosis, age, gender, tumor stage, tumor grade according to World Health Organization classification of tumors, PS based on The Eastern Cooperative Oncology Group (ECOG) score, investigations, clinical course, with details of concurrent chemotherapy, radiotherapy, nimotuzumab therapy [8,9].

Treatment protocol

Two treatment group were defined. Patients were randomized to receive the treatment by simple randomization method.

Nimotuzumab plus CRT group consisted of patients who received nimotuzumab chemoradiation therapy as follows: nimotuzumab 200 mg every week for six consecutive weeks; cisplatin 30 mg/m2 every week for six weeks; radiotherapy of 2-Gy/fraction, five fractions/week for a total dose of 70 Gy; neck lymph node invasion prophylaxis of 50 Gy. CRT group was treated with CRT (without nimotuzumab).

Follow-up during and after treatment

The treatment response was evaluated at eight weeks following radiotherapy using Response Evaluation Criteria in Solid Tumors version 1.0 [10]. The responses assessed included complete response (CR), partial response (PR), the progression of disease (PD), and stable disease (SD) based on computed tomography (CT) findings.

Evaluation of side effects was performed according to Common Terminology Criteria Standard for Adverse Events version 3.0 [11]. Patients were followed up for at least two years or death to assess survival time.

Statistical analysis

Data were processed using Statistical Package for the Social Sciences for Windows, Version 16.0. (SPSS Inc., Chicago, IL). Progression-free survival (PFS) and overall survival (OS) were measured using the Kaplan-Meier method from the beginning of treatment until the date of death or last date of follow-up. Univariate analysis of patient characteristics and tumor response was conducted by chi-square test and Fisher’s exact test. All probability values were two-sided, and P values <0.05 were considered statistically significant.

## Results

A total of 87 patients were included in the study; 43 patients were in the nimotuzumab plus CRT group and 44 in the CRT alone arm. Table 1 summarizes the characteristics of 87 patients included in the analysis. There was no significant difference between the two groups in terms of age, gender, tumor location, tumor/node/metastasis stage, grade, and PS of patients.

**Table 1:** Patient characteristics CRT: chemoradiotherapy.

Characteristics	Nimotuzumab plus CRT group	CRT alone group
Age group (n, %)		
30-39 years	4 (9.3)	2 (4.5)
40-49 years	9 (20.9)	9 (20.5)
50-59 years	21 (48.0)	25 (56.8)
≥ 60 years	9 (20.9)	8 (18.2)
Gender (n,%)		
Male	40 (93.0)	42 (95.5)
Female	3 (7.0)	2 (4.5)
Location (n,%)		
Oral cavity	11 (25.5)	15 (34.1)
Oropharynx	10 (23.3)	8 (18.1)
Hypopharynx, larynx	22 (51.2)	21 (47.8)
T-stage (n,%)		
1	0 (0.0)	0 (0.0)
2	1 (2.3)	1 (2.3)
3	13 (30.2)	16 (36.4)
4A	20 (46.5)	20 (45.5)
4B	9 (20.9)	7 (15.9)
N-stage (n,%)		
N0	13 (30.2)	16 (36.4)
N1	12 (27.9)	12 (27.3)
N2	12 (27.9)	12 (27.3)
N3	6 (14.0)	4 (9.1)
Overall Stage (n,%)		
III	7 (16.3)	14 (31.8)
IVa	22 (51.1)	19 (43.2)
IVb	14 (32.6)	11 (25.0)
Grade		
I	6 (14.0)	6 (13.6)
II	29 (67.4)	34 (77.3)
III	8 (18.6)	4 (9.1)
Performance status (PS) (n,%)		
0	26 (60.5)	21 (47.7)
1	13 (30.2)	18 (40.9)
2	4 (9.3)	5 (11.4)

The complete and partial response rates of nimotuzumab plus CRT group were 48.8% and 37.2%, respectively, compared to the 34.1% and 36.4% complete and partial response rates (respectively) in CRT group. There was no significant difference between the two groups (Table 2).

The overall tumor response rate across both groups was 80.5% (with 48.3% complete response and 32.2% partial response). The tumor response rate in the nimotuzumab plus CRT group and CRT alone arm was 90.6% and 70.4%, respectively; the difference was statistically significant (p = 0.029). Overall, the lymph node response across both groups was 82.8% (39.7% complete response and 43.1% partial response). The general response in the two groups was 78.2%, with 41.4% complete response and a 36.8% partial response (Table 2).

**Table 2:** Response rate within the two groups CRT: chemoradiotherapy.

Response	Nimotuzumab plus CRT groups	CRT alone group
Tumor (n,%)		
Complete	25 (58.1)	17 (38.6)
Partial	14 (32.5)	14 (31.8)
No response	2 (4.7)	9 (20.5)
Progress	2 (4.7)	4 (9.1)
Lymph nodes (n,%)		
Complete	14 (46.7)	9 (32.1)
Partial	11 (36.7)	14 (50.0)
No response	2 (6.6)	2 (7.1)
Progress	3 (10.0)	3 (10.8)
General response (n,%)		
Complete	21 (48.8)	15 (34.1)
Partial	16 (37.2)	16 (36.4)
No response	4 (9.3)	6 (13.6)
Progress	2 (4.7)	7 (15.9)

**Figure 1:**
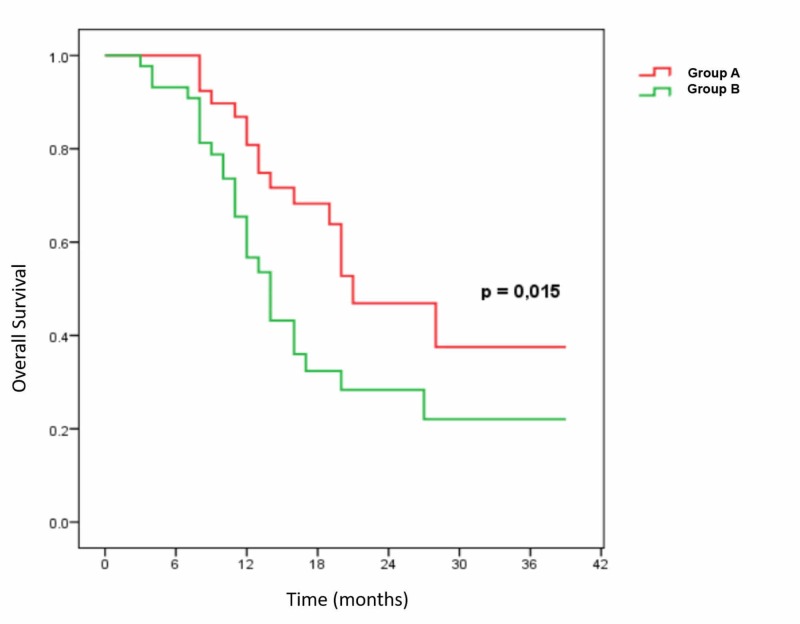
Overall survival of two groups Group A: Nimotuzumab plus chemoradiotherapy (CRT); Group B: CRT alone.

OS in the two groups at 12 and 24 months was 75.1% and 48.0%, respectively, in the nimotuzumab plus CRT group and 54.4% and 29.0% in CRT group; the difference was statistically significant (p = 0.016) (Figure 1).

Multivariable analysis identified lymph node metastasis as a risk factor affecting treatment outcomes when combining monoclonal antibody with chemoradiation therapy.

**Figure 2:**
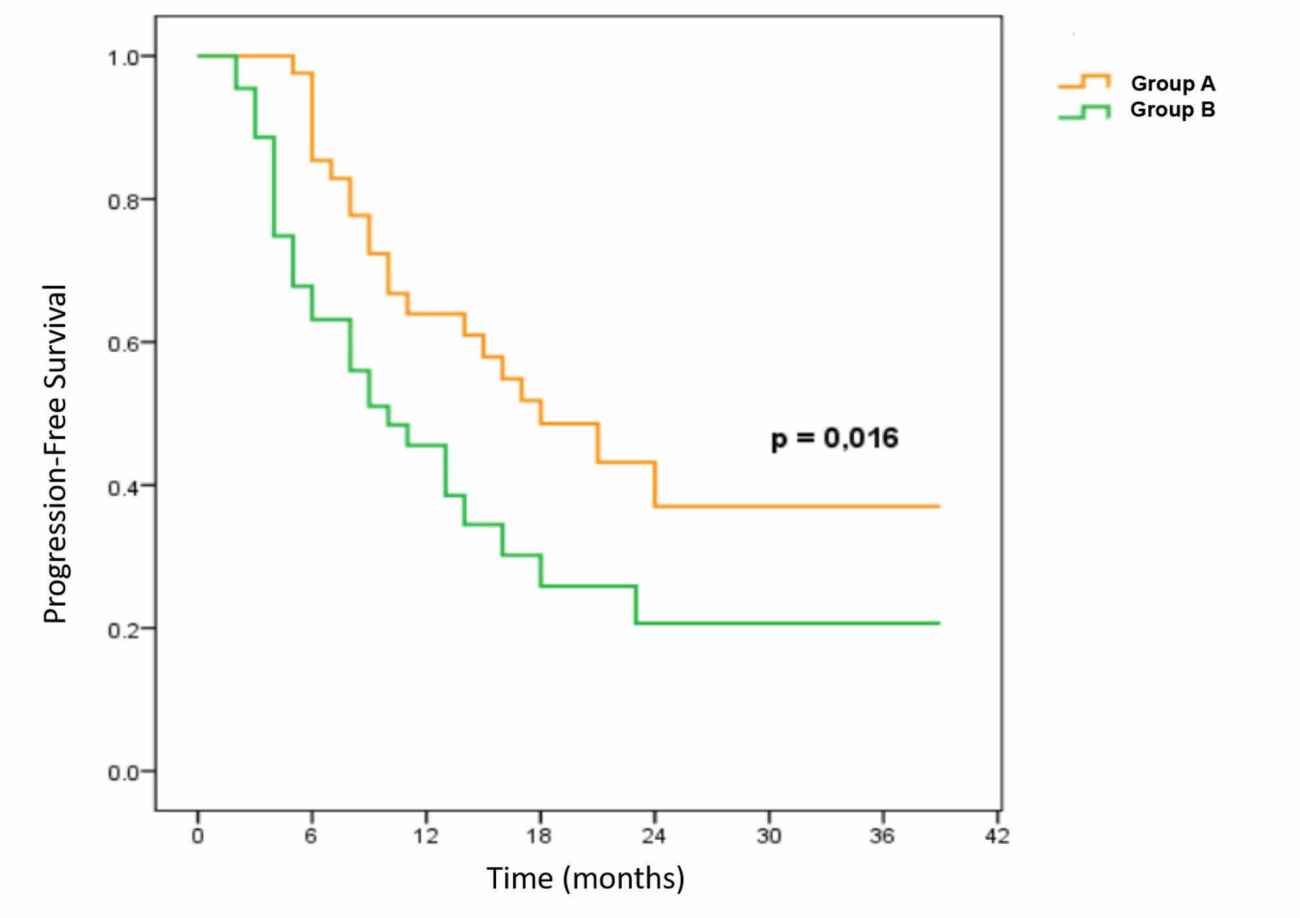
Progression-free survival of two groups Group A: Nimotuzumab plus chemoradiotherapy (CRT); Group B: CRT alone.

PFS of the two groups at 12 and 24 months was 64.2% and 37.4%, respectively, in the nimotuzumab plus CRT group compared to 39.5% and 21.3% in the CRT group. The difference was statistically significant (p = 0.016) (Figure 2).

The most common adverse events encountered during treatment and detailed toxicity and grade for the combination therapy are summarized in Table 3 and Table 4. Although these adverse events slightly delayed the treatment for several weeks, no patients halted therapy or died (Table 5).

**Table 3:** Infusion reactions CRT: chemoradiotherapy.

Side effects	Nimotuzumab plus CRT group (Number of patients)	CRT alone group (Number of patients)
Headache	2	0
Flushing	2	0
Itching	2	0
Myalgia	0	0
Hypotension	0	0
Chills	0	0
Fever	0	0
Shock	0	0
Total	43	44

**Table 4:** Other adverse events Group A: Nimotuzumab plus chemoradiotherapy (CRT); Group B: CRT alone; LFT: liver function test.

Side Effects	Stage I, II, III, IV			Stage III, IV		
	Group A (%)	Group B (%)	p	Group A (%)	Group B (%)	p
Neutropenia	9 (21.0)	14 (31.8)	>0.05	0 (0.0)	0 (0.0)	-
Anemia	13 (30.2)	17 (38.7)	>0.05	0 (0.0)	1 (2.3)	-
Thrombocytopenia	1 (2.3)	3 (6.8)	-	0 (0.0)	0 (0.0)	-
Nausea	16 (37.2)	16 (36.4)	>0.05	0 (0.0)	1 (2.3)	-
Vomiting	17 (39.5)	20 (45.5)	>0.05	0 (0.0)	1 (2.3)	-
Renal failure				1 (2.3)	0 (0.0)	-
LFT elevations	5 (11.6)	3 (6.8)	-	0 (0.0)	0 (0.0)	-
Mucositis	38 (88.4)	39 (88.6)	>0.05	11 (25.6)	9 (20.5)	>0.05
Dermatitis	26 (60.5)	22 (50.0)	>0.05	1 (2.3)	0 (0.0)	-
Rash	2 (4.7)	0 (0.0)	-	0 (0.0)	0 (0.0)	-

**Table 5:** Treatment delay due to side effects CRT: chemoradiotherapy.

Delay time	Nimotuzumab plus CRT group (n,%)	CRT alone group (n,%)
< 1 week	37 (86.0)	40 (90.1)
1–2 weeks	4 (9.3)	4 (9.1)
2–3 weeks	2 (4.6)	0 (0)
Stop treatment	0	0
Death	0	0
Total	43	44

## Discussion

We saw an overall tumor response rate across both groups of 80.5%, with 48.3% complete response and 32.2% partial response. Ramakrishnan et al. conducted a study among 40 patients treated with CRT with or without nimotuzumab, and they reported 34 cases had partial or complete response accounting for 85%, with complete response in 32 cases accounting for 80% of the cohort [5]. Their response rate was slightly higher than ours, with an obvious difference in the complete response rate. The difference in response rates may be because, in this study, the authors only took phase III, IVa (T1-4a, N0-2) and excluded T4B, N3, which constitute large and invasive tumors that spread, or indicates lymph nodes larger than 6 cm, thus signifying a difficult response to radiotherapy or CRT.

Several domestic studies also mentioned the response rate to CRT but only for specific tumor locations such as the lower pharynx larynx, or tonsils. According to Tung, tumor response rates were as high as 100% (complete and partial responses were 71.7% and 28.3% respectively), and lymph node response was also higher than in our study at 95.4% (62.8% complete and 32.6% partial response) (Ph.D. Thesis: Ngo Thanh Tung: Study on Clinical, Subclinical Characteristics and Results Chemistry - Accelerated Radiotherapy and Laryngeal Cancer in Stage (III - IVB) Cannot Be Operated at Vietnam Cancer Hospital. Ha Noi Medical University. 2011). In that study, response assessment was conducted immediately after treatment, with follow-up for four weeks after the end of treatment. In our study, the assessment was performed later in order to fully evaluate the effectiveness of radiotherapy. According to Hoang et al., tonsillar cancer had a high response after CRT with 80% partial and 19% complete response [12]. While this could be explained by the fact that in this study, patients were mainly in stage III or IVa, tonsil carcinoma is still considered to have good treatment outcomes among head and neck squamous cell carcinomas.

The anti-EGFR mAb incorporated into radiotherapy increased response rates across multiple studies. According to Rodríguez, the complete response among patients treated with nimotuzumab combined with radiotherapy was 59.5%, compared with 34.2% for radiotherapy-placebo. The difference was statistically significant (p = 0.028), which showed that the combination with nimotuzumab had a better response than isolated radiotherapy [6]. Similarly, in the study by Ramakrishnan et al., nimotuzumab increased complete response significantly when combined with radiotherapy (76% versus 37%, p = 0.023) [5].

Nimotuzumab also increased response rates when combined with CRT, as reported by Ramakrishnan et al. With 20 patients in each group, when combining with nimotuzumab, total response (including complete response and partial response) rose to 100%, compared with 70% in the group treated with radiotherapy alone (p = 0.02) [5]. Similarly, Patil et al. demonstrated that nimotuzumab in combination with cisplatin and radiotherapy was superior to cisplatin and radiotherapy in improving the PFS, duration of locoregional control and disease-free survival in locally advanced squamous head and neck cancer [13].

In our study, the tumor response rate in nimotuzumab plus CRT group and CRT alone arm was 90.6% and 70.4%, respectively; the difference was statistically significant (p = 0.029). The lymph node response rate and general response rate were higher in the nimotuzumab plus CRT group compared with the CRT group, but the difference was not statistically significant. The complete response rate was higher in the nimotuzumab plus CRT group, but the difference was not statistically significant. Because the number of patients in the study was limited, early results will need to be confirmed by further studies with larger sample sizes.

According to research results, the advantage observed in OS confirmed the benefits of anti-EGFR mAb combination. Specifically, OS at 12 months and 24 months was 75.1% and 48% in nimotuzumab plus CRT group respectively compared with 54.4% and 29% in CRT group; median survival was 20 months in nimotuzumab plus CRT group and 13 months in CRT group, a statistically significant difference (p = 0.015). This result was reproduced for PFS. The rate of PFS in the two groups at 12 and 24 months was 64.2% and 37.4% respectively in the nimotuzumab plus CRT group compared to 39.5% and 21.3% in CRT group; the difference between the two groups was statistically significant (p = 0.016).

Several large clinical trials have shown the effectiveness of anti-EGFR mAb in combination with radiotherapy or standard CRT. According to Bonner et al., cetuximab in combination with radiotherapy increased median OS from 29.3 to 49 months, decreased mortality rate by 27% (p = 0.018), total five-year survival from 36.4% to 45.6%, median PFS from 12.4 months to 17.1 months (p = 0.006); PFS at two years increased from 37% to 46% [3]. Rodriguez et al., when incorporating mAb nimotuzumab into radiotherapy, also found similar results with OS (mean and median) in the antibody nimotuzumab plus CRT group at 21.71 and 12.5 months versus placebo at 17.71 and 9.47 months [6]. This further supports the efficacy of the anti-EGFR mAb combination.

For anti-EGFR mAb combination with CRT, previous research has shown results consistent with ours, as demonstrated in a Phase II study by Ramakrishnan et al. showing two-year survival of 39.13% in the control group and 76.28% in the combination therapy group (p = 0.007). Mean survival was increased from 23.69 to 43.62 months when combining the treatment with antibodies [5]. Since patients with stage IVb disease were excluded, this study showed higher survival. However, the role of nimotuzumab in combination with standard CRT was also confirmed, similar to our study.

However, the effectiveness of anti-EGFR mAb CRT combination was not confirmed in a 40-month study by Ang et al., wherein 895 patients were evaluated, and initial results showed no difference in OS and PFS [14].

Hematopoietic system toxicity included neutropenia, anemia, thrombocytopenia, mainly at the level I and II. Anemia was most common (30.2% in nimotuzumab plus CRT group and 38.7% in CRT group). Only 2.3% of patients in the CRT group had anemia grade III. Neutropenia was only recorded in grade I and II and occurred in 21% of patients in the nimotuzumab plus CRT group and 31.8% in the CRT group. Thrombocytopenia was rare, occurring at a rate of 2.3% in the nimotuzumab plus CRT group and 6.8% in the CRT group. Hematological toxicity in the CRT group was most frequently observed in grade II, and anemia was the most common finding in our study and others [15,16]. In the study Tung, using cisplatin 100 mg/m2 every three weeks, the rate of hematopoietic system toxicity was higher than that observed in our study. However, the severity was relatively similar (Ph.D. Thesis: Tung, 2011).

The rate of vomiting in our study was lower than that reported by Hoang, with 51.5% of patients experiencing vomiting, the majority of which was grade I (45.3%), while grade II accounted for only 6.2%. The high rate of vomiting in this study may be due to high dose cisplatin (100 mg/m2 every three weeks) [12]. Although cisplatin is nephrotoxic, with low-dose weekly use, renal failure was rare and mild in our experience. Only one case of renal failure (grade III) was noted, accounting for 2.3% of patients in the CRT group; the toxicity was reversible, and the resulting treatment delay was minimal.

The infusion reaction associated with mAb nimotuzumab may occur in a small percentage of patients and can usually be fully controlled. According to Crombet et al., this undesirable effect occurs immediately after the administration of nimotuzumab [4]. Patients may experience fever, nausea, vomiting, high blood pressure, chills, headache, disorientation, chest pain, speech disturbances, and/or muscle aches. In our study, hypersensitivity reactions were infrequent, occurring in only two of 43 cases in the nimotuzumab plus CRT group and manifesting as headache, flushing, and itching (accounting for 4.7% for each type of effect). No cases of anaphylactic shock during infusion were reported. These results are in line with the conclusions of previous studies on the safety of nimotuzumab.

In our study, skin rash occurred in only two of the 43 patients in the nimotuzumab plus CRT group, accounting for 4.7%. Both reactions were rated as grade I and were controlled with a topical cream, without disturbing the course of therapy. This result was consistent with other studies using nimotuzumab.

## Conclusions

Nimotuzumab can be safely combined with chemoradiation therapy for locally advanced squamous cell carcinoma of the head and neck in order to achieve superior therapeutic response and improved survival outcomes without increased toxicity.
